# Overexpression of *OsPIN5b* Alters Plant Architecture and Impairs Cold Tolerance in Rice (*Oryza sativa* L.)

**DOI:** 10.3390/plants14071026

**Published:** 2025-03-25

**Authors:** Xiaoyu Fu, Guo Chen, Xinya Ruan, Guozhang Kang, Dianyun Hou, Huawei Xu

**Affiliations:** 1 College of Agriculture, Henan University of Science and Technology, Luoyang 471000, China; 13608423875@163.com (X.F.); cguo1010@163.com (G.C.); 19837068271@163.com (X.R.); dianyun518@163.com (D.H.); 2 National Key Laboratory of Wheat and Maize Crop Science, Henan Agricultural University, Zhengzhou 450046, China; guozhangkang@henau.edu.cn

**Keywords:** *OsPIN5b*, polar auxin transport, cold tolerance, ROS homeostasis, rice (*Oryza sativa* L.)

## Abstract

Auxin plays a versatile role in regulating plant growth and development. The auxin efflux carrier PIN-FORMED (PIN) proteins dictate the distribution and maximum of auxin within various tissues. Despite extensive research on OsPINs in recent years, their functions in abiotic stress resistance, particularly cold tolerance, remain poorly understood. Here, we investigated the role of *OsPIN5b* in rice (*Oryza sativa* L.) growth and development, as well as its contribution to cold tolerance using overexpression technology. Overexpression of *OsPIN5b* (OE) resulted in reduced shoot height and a lower number of adventitious roots at the seedling stage. Transgenic rice plants exhibited an earlier heading date, stunted growth, and compromised agronomic traits, including shortened panicle length, decreased grain number per panicle, reduced seed size, and lower seed setting rate during the reproductive stage. Auxin content in the transgenic lines was significantly elevated, as indicated by the upregulation of the auxin-responsive gene *OsIAA20* and increased auxin levels quantified using a newly developed method. Compared with wild-type plants, the cold tolerance of OE plants was markedly reduced, as evidenced by lower survival rates, higher levels of electrolyte leakage, and increased malondialdehyde (MDA) production following cold treatment. In line with this, the transgenic lines produced less soluble sugar and proline, while accumulating more hydrogen peroxide (H_2_O_2_) and superoxide anion radicals ($O_{2}^{-}$) after cold treatment. Furthermore, the activities of antioxidant enzymes, including catalase (CAT), superoxide dismutase (SOD), and peroxidase (POD), were notably decreased upon cold treatment compared with those in WT plants. Additionally, *OsRBOHH*, which plays a role in ROS production, was significantly upregulated in transgenic lines both before and after chilling stress, suggesting that *OsRBOHH* plays a potential role in regulating ROS production. Collectively, overexpression of *OsPIN5b* substantially disturbs auxin homeostasis, resulting in impaired plant architecture and agronomic traits. More importantly, the upregulation of *OsPIN5b* compromises rice cold tolerance by perturbing ROS homeostasis and adversely influencing the accumulation of soluble sugar and proline.

## 1. Introduction

Rice (*Oryza sativa* L.) is one of the most important crops worldwide, originating from tropical and subtropical regions. Compared to other cereal crops, rice exhibits greater sensitivity to cold stress [1,2,3,4]. Additionally, the increasing frequency of extreme weather events exposes plants to various abiotic stresses, including cold stress. Cold stress not only restricts the geographical distribution of plants but also significantly reduces their productivity [5,6]. For rice, cold stress primarily impairs growth and development during the early stages of seedling establishment [7], as well as at the booting stage [2,8]. Research has demonstrated that low temperatures can reduce rice yields by 30–40% in temperate regions [9]. Therefore, improving rice cold tolerance is essential for ensuring food security [10].

In recent decades, extensive research has been conducted on the molecular regulatory mechanisms underlying plant cold tolerance [11,12,13]. Notably, the well-known *C-repeat binding factor* (*CBF*)/*dehydration-responsive element binding factor* (*DREB*) genes play crucial roles in regulating plant cold tolerance [14,15,16]. Upregulation of *OsDREB1s*, such as *OsDREB1A*, *OsDREB1B*, *OsDREB1F*, and *OsDREB1G*, has been shown to significantly enhance rice cold tolerance [17,18,19,20]. In contrast, several studies have demonstrated that plant cold tolerance is not directly correlated with the expression levels of these genes [21,22,23], highlighting the complexity of the molecular mechanisms underlying plant cold adaptation. Additionally, it has been reported that Ca^2+^ signaling is involved in regulating plant cold adaptation. For example, COLD1 (CHILLING-TOLERANCE DIVERGENCE 1) and OsCNGC9 (CYCLIC NUCLEOTIDE-GATED CHANNEL 9) regulate rice cold tolerance by facilitating Ca^2+^ influx in response to cold stress [10]. Cold stress triggers the production of ROS and regulates plant cold tolerance [24,25]. Low levels of ROS, typically produced during the early stages of stress, function as signaling molecules to induce a variety of stress responses, thereby enhancing stress tolerance. In contrast, excessive accumulation of ROS at later stages of stress can cause cellular damage and severely impair plant growth and development [13,23]. However, the precise mechanisms by which plants modulate ROS homeostasis remain unclear. Additionally, numerous regulatory modules, such as SEC13 Homolog 1 (SEH1)-DREB1s-cold regulated (or responsive) genes (CORs), temperature-induced lipocalins (TIL1)-fatty acid desaturases (FADs), NAM, ATAF1/2, and CUC 5 (NAC5)-abscisic acid insensitive 5 (ABI5)-CORs, chilling-tolerance in Geng/japonica rice 3 (COG3)-filamentation temperature-sensitive H 2 (FtsH2)-D1, mitogen-activated protein kinase 6 (MAPK6)-inducer of CBF expression 1 (ICE1)/ideal plant architecture 1 (IPA1), histone deacetylase 716 (HDA716)-basic leucine zipper 46 (bZIP46)-DREB1A/COLD1, and COG1-somatic embryogenesis receptor kinases-like 2 (SERL2)-MAPK3, have been reported to be involved in rice cold tolerance [26,27,28,29,30,31,32].

As the first-discovered phytohormone, auxin not only regulates nearly all aspects of plant growth and development [33,34,35,36] but also plays a crucial role in mediating responses to multiple biotic and abiotic stresses, including cold stress [37,38]. Exogenous application of auxin enhances plant cold tolerance [39], and auxin levels significantly increase in response to cold treatment [40]. This findings suggest that auxin plays a crucial role in regulating plant cold tolerance. The biosynthesis, transport, conjugation, and catabolism of auxin can markedly influence auxin levels and spatial distribution within plants [41,42,43], which may subsequently modulate auxin signaling pathways and thereby affect cold tolerance. However, investigations have demonstrated that plant cold tolerance is likely linked to polar auxin transport (PAT) [44]. PAT is closely associated with auxin transport carriers, specifically the PIN-FORMED (PIN) proteins and influx carriers AUXIN RESISTANT1 (AUX1)/LAX family members [45,46]. Among these, PIN carriers play a particularly crucial role in PAT [47]. An earlier report demonstrated that low temperature can suppress auxin transport rate in plants [48]. Cold treatment inhibits basipetal auxin transport in the inflorescence stem, while room temperature restores it [49]. Cold stress primarily blocks the intracellular trafficking of PIN2 and PIN3, thereby inhibiting shootward auxin transport [44]. GNOM, a SEC7-domain-containing ARF-GEF that affects PAT and development in plants [50,51,52], has been shown to mediate the cold stress response in *Arabidopsis* [53]. In agreement with this, our studies showed that *OsPIN9* positively regulates rice cold tolerance [54,55], whereas the mutation of *OsPIN1b* impairs rice cold tolerance [56]. Collectively, accumulating evidence suggests that plant cold tolerance is closely associated with PAT, particularly the *PIN* genes. However, the detailed molecular mechanisms remain largely elusive.

The rice genome contains 12 *OsPIN* genes, and their tissue-specific expression patterns as well as responses to various hormones and abiotic stresses have been extensively investigated [57,58,59]. OsPIN5b is targeted to endoplasmic reticulum (ER) and plays a crucial role in regulating auxin homeostasis, transport, and distribution, thereby influencing rice plant architecture and yield [60]. However, the role of *OsPIN5b* in regulating rice cold tolerance remains unclear. In this study, we upregulated the expression of *OsPIN5b* and found that it regulates rice plant architecture and key agronomic traits, likely due to altered auxin homeostasis. Furthermore, we observed that overexpression of *OsPIN5b* significantly impairs rice cold tolerance, primarily due to the perturbation of ROS homeostasis and decline in the accumulation of soluble sugar and proline.

## 2. Results

### 2.1. Generation of OsPIN5b-Overexpressing Lines and Phenotypes of Transformants

**Figure 1:**
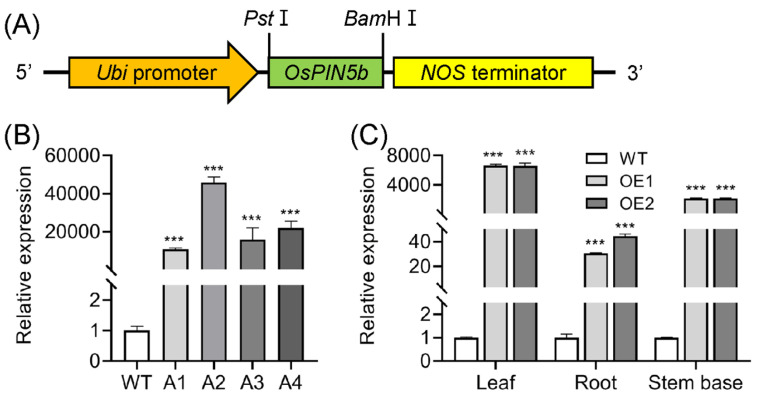
Generation of *OsPIN5*-Overexpressing lines: (**A**) schematic diagram of *pUbi*:*OsPIN5b*; (**B**) expression analysis of *OsPIN5b* in different transgenic lines; (**C**) expression analysis of *OsPIN5b* in various tissues of selected transgenic lines. Values are means ± standard deviation (SD) (*n* = 3). Data were analyzed by ANOVA and Tukey’s tests at a *p* < 0.05 significance level. ***: *p* < 0.001.

To investigate the role of *OsPIN5b* in regulating plant growth, development, and abiotic stress resistance, we employed overexpression technology to upregulate its expression. A plasmid harboring the *OsPIN5b* ORF under the control of a strong constitutive *Ubiquitin* promoter (*pUbi*) was introduced into the rice cultivar Nipponbare via *Agrobacterium*-mediated transformation (Figure 1A). The presence of the transgene in the resulting transgenic lines was confirmed by PCR using genomic DNA as the template. Compared with the WT plants, qRT-PCR analysis revealed that *OsPIN5b* was greatly elevated in different OE lines (Figure 1B). Among these, lines A3 and A4, which exhibited similar expression levels of *OsPIN5b* and were subsequently renamed as OE1 and OE2, were employed for further investigation. We then examined the expression of *OsPIN5b* in leaves, roots, and stem bases of these two transgenic lines. The highest increase in expression was observed in the leaves, followed by the stem bases, while the lowest expression was detected in the roots (Figure 1C). These results indicate that *OsPIN5b* expression is substantially increased in the transgenic lines.

We subsequently analyzed the phenotype of OE lines at the seedling stage. The plant height, root length, and number of adventitious roots in 7-day-old seedlings were comparable to those in WT plants (Figure S1A). However, as the plants continued to grow, consistent with previous findings on *OsPIN5b* overexpression [60], upregulation of *OsPIN5b* resulted in retarded growth in 14-day-old seedlings. Specifically, the shoot height of OE lines was significantly reduced compared with WT plants, and the number of adventitious roots in OE lines was notably lower than that in WT plants (Figure S1B). In contrast, the root length in the transgenic plants remained comparable to that of WT plants (Figure S1B), which differs from previous findings [60]. These results suggest that overexpression of *OsPIN5b* primarily affects shoot height and adventitious root growth rather than primary root growth, at least during the seedling stage.

Apart from altering the phenotype at the seedling stage, overexpression of *OsPIN5b* also significantly impaired rice growth and development at the mature stage. The plant height of the OE lines was reduced by approximately 20% compared with WT plants, primarily due to a decrease in internode length. Additionally, we observed that the heading date of OE lines occurred approximately one week earlier than that of WT plants (Figure S2).

In addition, the upregulation of *OsPIN5b* impaired panicle development and seed setting rate, consistent with previous findings [60]. Specifically, compared to WT plants, OE lines exhibited significant reductions in panicle length, number of branches per panicle, grain number per panicle, grain weight per panicle, and seed setting rate (Figure S3). These results suggest that the overexpression of *OsPIN5b* adversely affects these key agronomic traits.

### 2.2. Upregulation of OsPIN5b Disrupts Auxin Homeostasis

**Figure 2:**
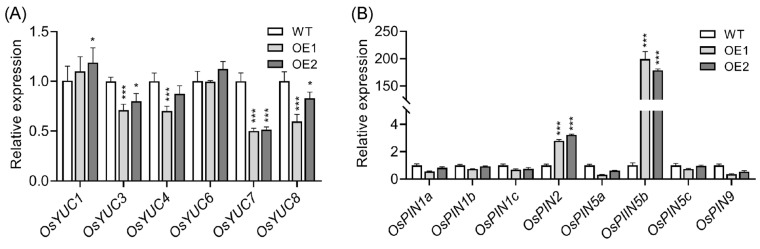
Expression analysis of *OsYUC* (**A**) and *OsPIN* (**B**) genes in wild-type and OE lines. Values are means ± standard deviation (SD) (*n* = 3). Data were analyzed by ANOVA and Tukey’s tests at a *p* < 0.05 significance level. *: *p* < 0.05; ***: *p* < 0.001.

To investigate the effect of upregulation of *OsPIN5b* on auxin homeostasis, we first examined the expression levels of *OsYUC* and *OsPIN* genes in both WT and OE roots. Several *OsYUC* genes, including *OsYUC3*, *OsYUC7*, and *OsYUC8*, displayed a significant decrease in expression in OE lines compared with WT plants, while the expression levels of other *OsYUC* genes remained comparable to those in WT plants (Figure 2A). Given that *OsYUC* genes are substantially upregulated under auxin deficiency, which suggests a potential metabolic compensation mechanism for auxin deficiency [61], it is plausible to infer that the depressed expression of *OsYUC* genes in OE lines may be attributed to elevated auxin levels. Furthermore, most *OsPINs* remained unchanged in OE lines compared with WT plants, with the exception of *OsPIN2* and *OsPIN5b* (Figure 2B). The high expression of the target gene *OsPIN5b* is reasonable, as its overexpression primarily leads to the upregulation of *OsPIN2*, suggesting that these two genes may coordinately regulate auxin transport in rice roots.

**Figure 3:**
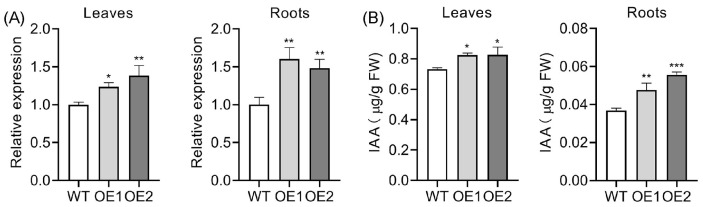
Upregulation of *OsPIN5b* increases auxin content in rice (*Oryza sativa* L.): (**A**) expression analysis of *OsIAA20* in WT and OE lines; (**B**) determination of auxin content in WT and OE lines. Values are means ± standard deviation (SD) (*n* = 3). Data were analyzed by ANOVA and Tukey’s tests at a *p* < 0.05 significance level. *: *p* < 0.05; **: *p* < 0.01; ***: *p* < 0.001.

Previous studies have demonstrated that *OsPIN5b* positively regulates free auxin content in rice leaves, roots, and panicles by facilitating the conversion of conjugated auxin forms to free auxin [60]. To investigate the auxin levels in OE lines, we utilized *OsIAA20*, a gene commonly employed as a marker for evaluating auxin content [62,63,64]. As expected, the expression of *OsIAA20* was significantly elevated in both OE leaves and roots (Figure 3A), suggesting that auxin levels likely increased due to the upregulation of *OsPIN5b*. Furthermore, we employed a newly developed method to measure auxin content in OE lines. Consistent with previous reports, auxin content was notably enhanced in OE leaves and root tips (Figure 3B). These results confirm that *OsPIN5b* indeed plays a critical role in regulating auxin homeostasis.

In addition, it is documented that the relative ratio of IAA-Asp to IAA decreases upon *OsPIN5b* upregulation [60]. Given the role of the GH3 family in regulating IAA homeostasis by conjugating amino acid (such as Asp, Ala, and Phe) to indole-3-acetic acid (IAA, the main form of auxin) [65], we further analyzed the expression of several *GH3* genes, which display a relative higher expression at the seedling stage [66], in both WT and OE lines. The results showed that most *GH3* genes displayed similar expression levels in WT and OE leaves, with the exception of *GH3-5* and *GH3-7*, which were significantly downregulated in OE lines compared with WT plants. Notably, *GH3-5* exhibited the highest expression among these genes (Figure S4A), implying that these two genes, particularly *GH3-5*, are likely involved in regulating auxin levels upon *OsPIN5b* overexpression. By contrast, several *GH3* genes, including *GH3-1*, *GH3-4*, *GH3-8*, and *GH3-9*, were significantly decreased in OE roots compared to WT roots (Figure S4B), implying that these genes may play a role in regulating auxin homeostasis in OE roots. Additionally, *GH3-5*, which was upregulated in OE leaves relative to WT plants, exhibited a substantial increase in OE roots. These findings indicate that rice leaves and roots likely possess distinct regulatory mechanisms for auxin homeostasis mediated by *GH3* genes.

### 2.3. Overexpression of OsPIN5b Impairs Rice Cold Tolerance

**Figure 4:**
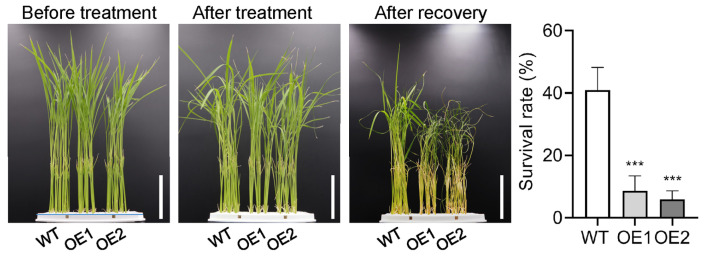
Overexpression of *OsPIN5b* impairs rice cold tolerance. Bar = 5 cm. Values are means ± standard deviation (SD) (*n* = 3). Data were analyzed by ANOVA and Tukey’s tests at a *p* < 0.05 significance level. ***: *p* < 0.001.

Previously, we demonstrated that low temperature conditions increase the expression of *OsPIN5b* [57], implying that *OsPIN5b* may play a potential role in regulating cold tolerance. To assess the effect of overexpression of *OsPIN5b* on rice cold tolerance, 14-day-old seedlings were transferred from 30 °C to 4 °C. After cold exposure for 5 days, seedlings were returned to room temperature for an additional 4 days. Approximately half of the WT plants leaves remained normal, whereas nearly all leaves of OE plants displayed rolling and wilting (Figure 4). Survival rate analysis revealed that overexpression of *OsPIN5b* significantly reduced rice cold tolerance. The survival rate of WT plants was approximately 40%, while it was less than 10% in the OE lines. These results indicate that OE plants are more sensitive to low temperatures compared to WT plants.

**Figure 5:**
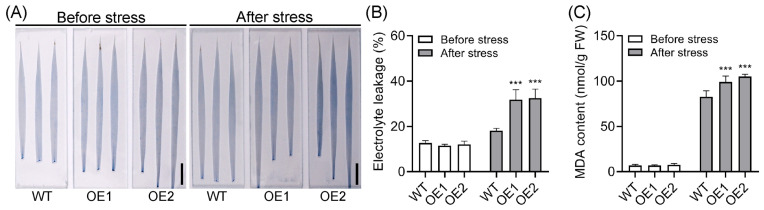
Upregulation of *OsPIN5b* results in cell damage: (**A**) trypan blue staining (Bar = 1 cm); (**B**) electrolyte leakage; (**C**) malondialdehyde (MDA) contents. Values are means ± standard deviation (SD) (*n* = 3). Data were analyzed by ANOVA and Tukey’s tests at a *p* < 0.05 significance level. ***: *p* < 0.001.

Cell death, electrolyte leakage, and malondialdehyde (MDA), were detected to further evaluate the cold tolerance of the OE lines. Trypan blue staining, a well-established method for assessing cell viability and membrane integrity, was employed to evaluate cellular damage. No apparent difference in trypan blue staining was observed between WT and OE leaves before cold stress. However, after 2 days of cold treatment, the intensity of trypan blue staining in OE leaves was notably darker than that in WT plants, particularly in OE2 plants, which exhibited a markedly lower survival rate following recovery (Figure 5A). This suggests that the cell death is more pronounced in the OE lines compared to WT plants. Low temperatures typically cause damage to cell membranes, as evidenced by electrolyte leakage, which is a hallmark of such damage [54,67]. In accordance with trypan blue staining, the electrolyte leakage in OE lines was comparable to that in WT plants under normal conditions but significantly higher than that in WT plants after cold stress treatment (Figure 5B). Consistently, cold stress also led to a marked increase in MDA content in OE lines compared with WT plants (Figure 5C). Collectively, these results strongly suggest that overexpression of *OsPIN5b* substantially impairs rice cold tolerance.

### 2.4. Soluble Sugar and Proline Content Decreased in OE Lines After Cold Treatment

**Figure 6:**
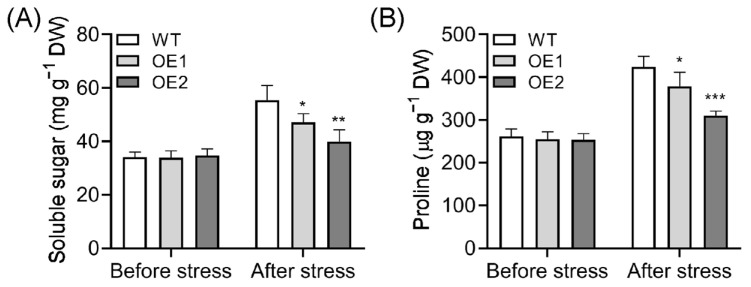
Soluble sugar (**A**) and proline (**B**) content measurement in WT and OE lines. Values are means ± standard deviation (SD) (*n* = 3). Data were analyzed by ANOVA and Tukey’s tests at a *p* < 0.05 significance level. *: *p* < 0.05; **: *p* < 0.01; ***: *p* < 0.001.

Abiotic stresses frequently trigger the accumulation of osmotic regulators, such as soluble sugars and proline, to enhance resistance against environmental stressors [68,69,70]. To evaluate the cold tolerance of *OsPIN5b*-overexpressing plants, we measured the levels of soluble sugars and proline. Prior to cold treatment, the content of soluble sugars and proline in OE lines was comparable to that in WT plants. However, after 48 h of cold stress, the levels of these osmotic regulators significantly decreased in OE lines compared to those in WT plants (Figure 6). These findings indicate that the lower levels of soluble sugars and proline in OE lines may contribute to decreased cold tolerance following exposure to cold stress.

### 2.5. Overexpression of OsPIN5b Disturbs ROS Homeostasis

**Figure 7:**
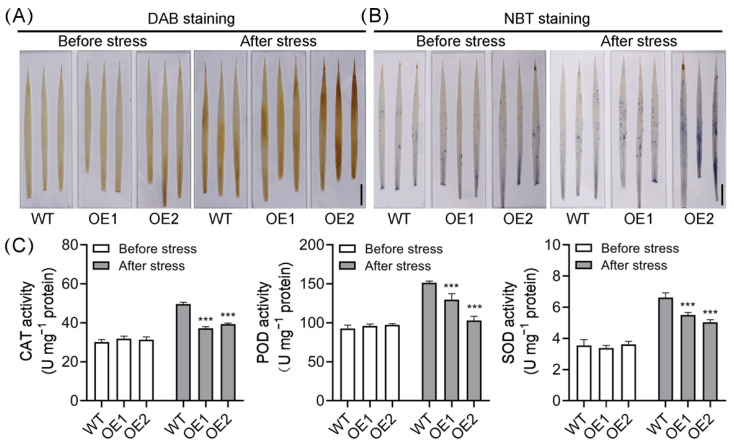
Overexpression of *OsPIN5b* disturbs ROS homeostasis: (**A**) DAB staining (Bar = 1 cm); (**B**) NBT staining (Bar = 1 cm); (**C**) measurement of CAT, POD and SOD activities. Values are means ± standard deviation (SD) (*n* = 3). Data were analyzed by ANOVA and Tukey’s tests at a *p* < 0.05 significance level. ***: *p* < 0.001.

Alongside the well-known CBF/DREB regulon, reactive oxygen species (ROS) also play a vital role in cold stress adaptation [23,54,55]. We then employed diaminobenzidine tetrahydrochloride (DAB 4HCl) staining and NBT staining to detect H_2_O_2_ and $O_{2}^{-}$, respectively, in WT and OE leaves before and after cold stress. No obvious difference was observed between WT and OE leaves before cold stress, while after cold stress, the OE leaves accumulated obviously more H_2_O_2_ and $O_{2}^{-}$ compared to WT plants (Figure 7A,B), indicating that the ROS homeostasis is disrupted in OE lines. ROS homeostasis is tightly associated with antioxidant enzyme activities, such as superoxide dismutase (SOD), catalase (CAT), and peroxidase (POD), which play vital roles in ROS scavenging [13,25]. We then measured these enzyme activities in WT and OE lines before and after cold stress. The activities of these three enzymes in OE lines were comparable to those in WT plants before cold stress. However, following cold stress treatment, the enzyme activities in OE lines were greatly suppressed (Figure 7C). Taken together, these results suggest that overexpression of *OsPIN5b* disrupts ROS homeostasis in rice, likely due to reduced antioxidant enzyme activities, thereby leading to cellular damage and impaired cold tolerance.

### 2.6. OsRBOHH Likely Plays a Role in ROS Production in OsPIN5-Overexpressing Lines

**Figure 8:**
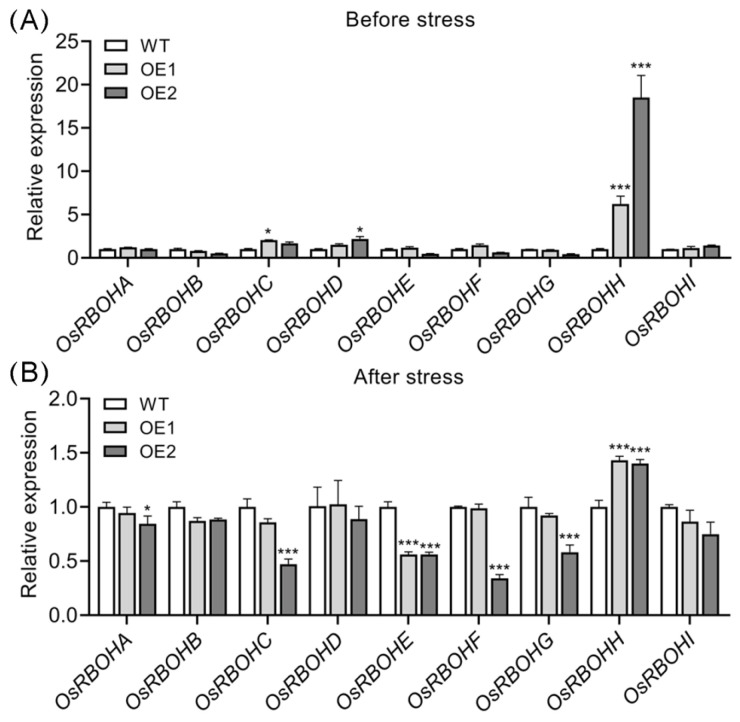
Expression analysis of *OsRBOH* genes in wild-type (WT) and *OsPIN5b*-overexpressing (OE) lines before (**A**) and after (**B**) cold treatment. The expression level of *OsRBOH* genes in WT was set as one. Values are means ± standard deviation (SD) (*n* = 3). Data were analyzed by ANOVA and Tukey’s tests at a *p* < 0.05 significance level. *: *p* < 0.05; ***: *p* < 0.001.

Plasma membrane-localized respiratory burst oxidase homologs (RBOH) are crucial for regulating ROS production and play a pivotal role in diverse cellular activities and responses to various abiotic and biotic stresses [71,72,73]. To date, nine *OsRBOH* genes have been identified in the rice genome [74,75,76]. Given that ROS homeostasis is substantially disrupted in OE lines, we investigated the expression of *OsRBOH* genes before and after cold stress. Prior to cold stress, only *OsRBOHH* was significantly upregulated in OE lines compared to WT plants, while the expression of other *OsRBOH* genes remained largely unchanged (Figure 8A). Upon cold treatment, however, only *OsRBOHE* showed a downward trend in expression in two OE lines, whereas *OsRBOHH* still exhibited a significant upregulation in OE lines relative to WT plants (Figure 8B). Considering the pronounced accumulation of ROS in OE lines after cold stress, it is plausible that *OsRBOHH* plays a vital role in modulating ROS production under cold stress conditions in these lines.

## 3. Discussion

Low temperature is a critical factor influencing plant growth and development [5,6]. In recent decades, numerous functional genes and regulatory modules have been identified as key players in regulating plant cold tolerance [12,77,78]. Additionally, phytohormones such as brassinosteroids (BR), ethylene, jasmonic acid (JA), abscisic acid (ABA), and auxin also play vital roles in plant cold tolerance [37,38,79]. Although the role of auxin in regulating plant cold tolerance has been established for decades [48], the underlying molecular mechanisms remain largely unknown. In this study, we investigated the function of an endoplasmic reticulum (ER)-localized protein, OsPIN5b, in rice growth and development, as well as its role in regulating cold tolerance through overexpression.

The rice genome possesses 12 *OsPIN* genes [58,59]. Bioinformatic analysis revealed that three *OsPIN* genes, namely, *OsPIN9*, *OsPIN10a*, and *OsPIN10b*, are specific to monocots [58]. Previous studies have investigated the roles of several *OsPIN* genes in regulating rice growth and development. For instance, RNAi-mediated suppression of *OsPIN1b* expression resulted in increased tiller angles, reduced plant height, and fewer adventitious roots [80]. Further analysis revealed that *OsPIN1a* and *OsPIN1b* exhibit redundant functions in rice development, whereas *OsPIN1c* and *OsPIN1d* play redundant roles in panicle formation [81]. *OsPIN2* is predominantly expressed in the root and shoot base, and functions in determining root system architecture and root gravitropism [82,83,84,85]. The *OsPIN5* subfamily comprises three genes: *OsPIN5a*, *OsPIN5b*, and *OsPIN5c* [58]. To date, only *OsPIN5b* was reported and functions in regulating rice architecture and yield [60]. Overexpression of *OsPIN5b* increases auxin levels across entire rice plants and impairs rice growth and development. In contrast, suppression of *OsPIN5b* by RNAi technology reduces auxin levels, promotes rice growth and development, and ultimately improves panicle length and grain yield. These findings suggest the potential significance of *OsPIN5b* in rice molecular breeding. Based on this finding, another report demonstrated that simultaneous mutation of three genes, *OsPIN5b*, *GS3* (a grain size gene), and *OsMYB30* (a cold tolerance gene), using CRISPR/Cas9 technology improves both rice yield and cold tolerance [86], further reinforcing the role of *OsPIN5b* in regulating rice grain yield. To investigate the role of *OsPIN5b*, we generated *OsPIN5b*-overexpressing rice plants (Figure 1). Consistent with previous findings [60], gene expression and auxin content measurements demonstrated that overexpression of *OsPIN5b* significantly disrupts auxin homeostasis (Figure 2 and Figure 3). Furthermore, *OsGH3-5* and *OsGH3-7* appear to play a crucial role in regulating this process (Figure 3). However, the mechanism by which the upregulation of *OsPIN5b* modulates the expression of *OsGH3* genes requires further investigation. In addition to altering the architecture at the seedling stage (Figure S1), overexpression of *OsPIN5b* also impairs panicle development and reduces the seed setting rate (Figure S3), suggesting that auxin homeostasis is critical for regulating rice agronomic traits. Consistently, loss-of-function mutations in the rice YUCCA (YUC) flavin-containing monooxygenase encoding genes *OsYUC2* or *OsYUC11* result in decreased auxin levels, leading to reduced panicle length, seed setting rate, seed size, and grain weight [87,88]. Additionally, research has shown that increased auxin levels due to mutations in the *dioxygenase for auxin oxidation* (*DAO*) gene can cause male sterility [89]. These findings strongly suggest that auxin levels during the reproductive stage must be tightly regulated for normal growth and development. The precise molecular mechanisms underlying auxin homeostasis at this stage warrant further investigation.

Auxin is involved in modulating plant cold tolerance, with polar auxin transport (PAT) likely playing a key role in this process [44]. Given that PIN carriers are crucial for controlling PAT, we hypothesized that *PIN* genes may be involved in modulating cold tolerance by regulating auxin homeostasis. To test this hypothesis, we investigated the cold tolerance of both wild-type and transgenic lines under cold stress conditions. Our findings indicated that the overexpression of *OsPIN5b* compromises rice cold tolerance. Compared to WT plants, the survival rate was significantly reduced in OE lines (Figure 4). Consistent with this observation, cell death, electrolyte leakage, and MDA content were markedly increased in OE lines relative to WT plants (Figure 5). Additionally, two critical osmolytes, soluble sugars and proline, were substantially decreased in OE lines (Figure 6). This is consistent with previous research showing that the tomato (*Solanum lycopersicum*) transcription factor SlWRKY51 enhances cold tolerance by promoting proline accumulation [70], underscoring the importance of proline in low-temperature adaptation. However, the molecular mechanisms underlying the decrease in proline levels in OE lines under low-temperature conditions require further investigation.

Despite the key role of auxin in regulating plant cold adaptation [39], the underlying molecular mechanisms remain largely unknown. Previous reports have demonstrated that OsPIN9 is localized to the plasma membrane and plays a crucial role in regulating auxin distribution in both rice shoots and roots [90]. Our research further revealed that *OsPIN9* negatively regulates cold tolerance in rice [54,55]. These results suggest that the spatial distribution of auxin in different tissues likely plays a critical role in regulating rice cold tolerance. By contrast, OsPIN5b is localized to the ER, and modulation of *OsPIN5b* expression not only alters auxin levels throughout the entire plant by influencing auxin conjugation [60] but also potentially affects the intracellular distribution of auxin, which may contribute to changes in rice cold tolerance (Figure 4). Therefore, it appears that OsPIN5b regulates rice cold tolerance by finely modulating the homeostasis between free auxin and conjugated auxin within cells, rather than by mediating auxin transport between different tissues. Collectively, these results indicate that both the spatial distribution of auxin across different tissues and the homeostasis of free auxin and conjugated auxin within cells likely play pivotal roles in regulating rice cold tolerance. While the molecular mechanism underlying auxin-mediated regulation of plant cold tolerance warrants further investigation.

ROS homeostasis plays a crucial role in regulating plant cold tolerance [23]. Accumulating evidence indicates that excessive ROS production under abiotic stress is a primary factor leading to cellular damage in plants [23,24,91,92,93]. For instance, a point mutation in the *low-temperature tolerance 1* (*LTT1*) gene enhances rice cold tolerance by activating ROS metabolic systems [8]. An APETALA2/ethylene-responsive factor (ERF) transcription factor OsERF096 negatively regulates rice cold tolerance by suppressing ROS scavenging [94]. In rice, compared to the *indica* varieties, the *japonica* varieties generally exhibit higher cold tolerance. This may be attributed to their faster accumulation of ROS during the early cold stage and lower ROS accumulation during recovery stages [23]. Our previous study demonstrated that *OsPIN9*, a monocot-specific auxin efflux carrier gene in rice negatively regulates cold tolerance, and ROS homeostasis plays a crucial role in the cold response process [54,55]. These findings suggest that auxin is likely linked to ROS regulation and is involved in modulating cold tolerance. In line with this, overexpression of *OsPIN5b* decreased ROS scavenging ability and resulted in ROS accumulation under cold stress (Figure 7), highlighting the critical role of ROS homeostasis during cold treatments. Additionally, proline plays a crucial role in the antioxidative network that alleviates stress-induced oxidative damage [95]. Our results showed that proline content was significantly reduced in OE lines under cold stress conditions (Figure 6). Consequently, the decreased proline content in OE lines may further contribute to ROS accumulation under low-temperature conditions. Previously, we demonstrated that the mutation of *OsPIN9* increased auxin levels in rice leaves, which subsequently triggered rapid ROS accumulation and contributed to enhanced cold tolerance [54]. Conversely, despite overexpression of *OsPIN5b* also increasing auxin levels (Figure 3), it impaired cold tolerance and ROS scavenging in OE lines (Figure 4 and Figure 7). These results suggest that a distinct regulatory mechanism may govern ROS homeostasis, underscoring the complexity of the molecular processes underlying rice cold adaptation. Additionally, we observed that *OsRBOHH* was significantly upregulated in OE lines under both normal and low-temperature conditions (Figure 8), indicating its potential role in ROS production in these lines. Given the extremely low expression levels of *OsRBOHH* in rice roots, leaves, shoots, and calli [74], we suspect that *OsRBOHH* likely plays a primary role under abiotic stress conditions, particularly under cold stress. However, the mechanism by which *OsPIN5b* regulates *OsRBOHH* requires further investigation. To elucidate the genetic relationship between *OsPIN5b* and *OsRBOHH*, investigation of *OsRBOHH* mutation in *OsPIN5b*-overexpressing lines and assessment of ROS accumulation following cold treatment could potentially provide more conclusive evidence. By contrast, a recent report demonstrated that *OsRBOHI* is essential for ROS production in rice [96]. However, in our study, the expression levels of *OsRBOHI* in OE lines were comparable to those in WT plants both before and after cold stress (Figure 8), suggesting that *OsRBOHI* may play a limited role in ROS production in OE lines.

## 4. Materials and Methods

### 4.1. Plant Materials, Growth Conditions and Cold Treatment

The rice *japonica* variety “Nipponbare” was utilized for the physiological experiments and genetic transformation. Hydroponic experiments were conducted following the method described in our previous study [97]. Briefly, rice seeds were surface-sterilized and subsequently cultured in darkness at 30 °C for 3 to 4 days. The germinated seeds were then transferred to Kimura B complete nutrient solution in plant growth chambers. The chambers were set to maintain a photoperiod of 12 h light (30 °C) and 12 h darkness (25 °C), with a relative humidity of 60–70%.

To evaluate the cold tolerance of rice seedlings, 14-day-old seedlings were transferred to low-temperature conditions (4 °C) for 5 days and then allowed by a recovery period under normal conditions. Subsequently, the survival rate was analyzed.

### 4.2. Vector Construction and Generation of the Transgenic Plants

PrimeSTAR HS DNA Polymerase (Takara Biotechnology Co., Ltd., Dalian, China) was employed to amplify full-length *OsPIN5b* from rice cDNA using specific primers listed in Table S1. The plant expression vector pCAMBIA1301-pUbi was kindly provided by Dr. Yao-Guang Liu (College of Life Sciences, South China Agricultural University, Guangzhou, China). The *OsPIN5b* gene was inserted into pCAMBIA1301-pUbi at the *Pst* I and *Bam*H I restriction sites. The resulting recombinant vector was introduced into rice variety “Nipponbare” following a previously described protocol [98]. Two homozygous transgenic lines, OE1 and OE2, confirmed by PCR and Hygromycin B screening, were used for further investigation.

### 4.3. Measurement of Auxin Levels

In addition to gene expression analysis, we employed a recently developed method, with slight modifications, to quantify auxin levels in rice leaves and roots as described in a previous study [99]. Briefly, after a 7-day germination period, approximately 0.05 g of seedling leaves or roots was collected and thoroughly homogenized using a multi-sample tissue grinder (Tiss-Basic48, Shanghai Jingxin Industrial Development Co., Ltd., Shanghai, China) in the presence of liquid nitrogen. Subsequently, 100% ethanol (0.3 mL) was added to the powdered sample, and the mixture was then centrifuged at 12,000 rpm for 10 min at 4 °C. Next, 100 μL of the supernatant was mixed with 900 μL of assay reagent (composed of water, concentrated sulfuric acid, and 0.5 M FeCl_3_ in a ratio of 25 : 15 : 0.75) and incubated at room temperature for 30 min. The absorbance of the resulting reaction mixture was measured at 540 nm and used for auxin content determination.

### 4.4. Quantitative Real-Time PCR (qRT-PCR) Analysis

qRT-PCR was performed according to our previously published protocol [57]. At least three biological replicates and three technical replicates were performed to assess gene expression levels. The *OsACTIN1* gene (Os03g0718100) served as the internal control. All primers utilized in this study are summarized in Table S1, and the gene names and ID numbers used for qRT-PCR analysis are provided in Table S2.

### 4.5. Physiological Analysis

Cell death was analyzed using trypan blue staining as previously reported [55,100]. Cell membrane integrity was assessed by measuring electrolyte conductivity and MDA content, following the methods described in a previous report [101]. For the determination of soluble sugar and proline content, 14-day-old seedlings were exposed to low-temperature conditions for 48 h. Subsequently, samples were collected and analyzed using the anthrone method for soluble sugar and the sulfosalicylic acid method for proline in accordance with previously established protocols [102,103]. 3,3′-diaminobenzidine (DAB) and nitro blue tetrazolium (NBT) staining were conducted following previously established protocols [23]. Briefly, rice leaves were incubated in DAB and NBT solutions, respectively, for 24 h at 37 °C. Subsequently, the samples underwent decolorization using 95% ethanol. The activities of catalase (CAT), peroxidase (POD), and superoxide dismutase (SOD) were measured according to previously described methods [104]. Protein content was quantified using the Coomassie Brilliant Blue G-250 staining method as described previously [105].

### 4.6. Statistical Analysis

Each experiment was independently replicated at least three times. Statistical analysis of the experimental data was conducted using one-way analysis of variance (ANOVA) via GraphPad Prism version 8.0.2 (GraphPad Software Inc., San Diego, CA, USA). Significance levels were set at *p* < 0.05 (*), *p* < 0.01 (**), and *p* < 0.001 (***). All data are presented as means ± standard deviation (SD).

## 5. Conclusions

Collectively, in this study, we elucidate the role of *OsPIN5b* in regulating rice architecture and agronomic traits, with a particular emphasis on its function in modulating cold tolerance. Our findings demonstrate that upregulation of *OsPIN5b* disrupts auxin homeostasis, which adversely affects plant growth and development. More importantly, upregulation of *OsPIN5b* significantly impairs rice cold tolerance, likely by suppressing ROS scavenging and reducing levels of soluble sugars and proline. Several issues warrant further investigation. For instance, the mechanism by which the expression levels of *OsPIN5b* influence auxin conjugation requires elucidation. Additionally, it is important to explore how auxin homeostasis regulates ROS homeostasis. Furthermore, whether *OsRBOHH* plays a vital role in regulating ROS production under cold stress conditions should be examined. Further investigation into these detailed issues may provide insights into the underlying molecular mechanisms of rice cold tolerance and identify potential targets for breeding cold-tolerant crops.

## Data Availability

The original contributions presented in this study are included in the article/Supplementary Materials. Further inquiries can be directed to the corresponding author(s).

## Supplementary Materials

The following supporting information can be downloaded at https://www.mdpi.com/article/10.3390/plants14071026/s1, Figure S1. Phenotypes of wild-type (WT) and OsPIN5b-overexpressing lines (OE) at 7 days (A) and 14 days (B) post-germination. Bar = 5 cm. Values are means ± standard deviation (SD; *n* = 30). Data were analyzed by ANOVA and Tukey’s tests at *p* < 0.05 significant level. *: *p* < 0.05; **: *p* < 0.01; ***: *p* < 0.001; Figure S2. Overexpression of *OsPIN5b* results in earlier heading date and retarded rice growth. (A) Phenotype at the headting stage. Bar = 10 cm. (B) The internode length analysis between WT and transgenic lines. Bar = 10 cm. Values are means ± standard deviation (SD; *n* = 18). Data were analyzed by ANOVA and Tukey’s tests at *p* < 0.05 significant level. *: *p* < 0.05; ***: *p* < 0.001; Figure S3. Overexpression of *OsPIN5b* influences rice agronomic traits. (A) Phenotype of wild-type (WT) and OE panicles. Bar = 4 cm. (B) Panicle length. (C) Branch number per panicle. (D) Grain number per panicle. (E) Grain weight per panicle. (F) Seed setting rate. Values are means ± standard deviation (SD; *n* = 38). Data were analyzed by ANOVA and Tukey’s tests at *p* < 0.05 significant level. *: *p* < 0.05; ***: *p* < 0.001; Figure S4. Expression analysis of *GH3* genes in WT and OE leaves (A) and roots (B). Values are means ± standard deviation (SD; *n* = 3). Data were analyzed by ANOVA and Tukey’s tests at *p* < 0.05 significant level. *: *p* < 0.05; ***: *p* < 0.001; Table S1: Primers used in this study; Table S2: Gene name and ID numbers used for qRT-PCR in this study.

## References

[1] Wang W., Vinocur B., Altman A. (2003). Plant responses to drought, salinity and extreme temperatures: Towards genetic engineering for stress tolerance. Planta.

[2] Zhang Q., Chen Q., Wang S., Hong Y., Wang Z. (2014). Rice and cold stress: Methods for its evaluation and summary of cold tolerance-related quantitative trait loci. Rice.

[3] Wang D., Liu J.L., Li C.G., Kang H.X., Wang Y., Tan X.Q., Liu M.H., Deng Y.F., Wang Z.L., Liu Y. (2016). Genome-wide association mapping of cold tolerance genes at the seedling stage in rice. Rice.

[4] Chen N., Xu Y., Wang X., Du C., Du J., Yuan M., Xu Z., Chong K. (2011). OsRAN2, essential for mitosis, enhances cold tolerance in rice by promoting export of intranuclear tubulin and maintaining cell division under cold stress. Plant Cell Environ..

[5] Ding Y., Shi Y., Yang S. (2020). Molecular regulation of plant responses to environmental temperatures. Mol. Plant.

[6] Shi Y., Ding Y., Yang S. (2018). Molecular regulation of CBF signaling in cold acclimation. Trends Plant Sci..

[7] Cheng C., Yun K.Y., Ressom H.W., Mohanty B., Bajic V.B., Jia Y., Yun S.J., de los Reyes B.G. (2007). An early response regulatory cluster induced by low temperature and hydrogen peroxide in seedlings of chilling-tolerant japonica rice. BMC Genom..

[8] Xu Y., Wang R., Wang Y., Zhang L., Yao S. (2020). A point mutation in *LTT1* enhances cold tolerance at the booting stage in rice. Plant Cell Environ..

[9] Andaya V.C., Mackill D.J. (2003). Mapping of QTLs associated with cold tolerance during the vegetative stage in rice. J. Exp. Bot..

[10] Ma Y., Dai X., Xu Y., Luo W., Zheng X., Zeng D., Pan Y., Lin X., Liu H., Zhang D. (2015). COLD1 confers chilling tolerance in rice. Cell.

[11] Ding Y., Yang S. (2022). Surviving and thriving: How plants perceive and respond to temperature stress. Dev. Cell.

[12] Guo X., Liu D., Chong K. (2018). Cold signaling in plants: Insights into mechanisms and regulation. J. Integr. Plant Biol..

[13] Zhang J., Li X.M., Lin H.X., Chong K. (2019). Crop improvement through temperature resilience. Annu. Rev. Plant Biol..

[14] Ding Y., Shi Y., Yang S. (2019). Advances and challenges in uncovering cold tolerance regulatory mechanisms in plants. New Phytol..

[15] Stockinger E.J., Gilmour S.J., Thomashow M.F. (1997). *Arabidopsis thaliana CBF1* encodes an AP2 domain-containing transcriptional activator that binds to the C-repeat/DRE, a cis-acting DNA regulatory element that stimulates transcription in response to low temperature and water deficit. Proc. Natl. Acad. Sci. USA.

[16] Liu Q., Kasuga M., Sakuma Y., Abe H., Miura S., Yamaguchi-Shinozaki K., Shinozaki K. (1998). Two transcription factors, DREB1 and DREB2, with an EREBP/AP2 DNA binding domain separate two cellular signal transduction pathways in drought- and low-temperature-responsive gene expression, respectively, in Arabidopsis. Plant Cell.

[17] Ito Y., Katsura K., Maruyama K., Taji T., Kobayashi M., Seki M., Shinozaki K., Yamaguchi-Shinozaki K. (2006). Functional analysis of rice DREB1/CBF-type transcription factors involved in cold-responsive gene expression in transgenic rice. Plant Cell Physiol..

[18] Wang Q., Guan Y., Wu Y., Chen H., Chen F., Chu C. (2008). Overexpression of a rice *OsDREB1F* gene increases salt, drought, and low temperature tolerance in both *Arabidopsis* and rice. Plant Mol. Biol..

[19] Moon S.J., Min M.K., Kim J.A., Kim D.Y., Yoon I.S., Kwon T.R., Byun M.O., Kim B.G. (2019). Ectopic expression of *OsDREB1G*, a member of the OsDREB1 subfamily, confers cold stress tolerance in rice. Front. Plant Sci..

[20] Zhang M., Zhao R., Huang K., Huang S., Wang H., Wei Z., Li Z., Bian M., Jiang W., Wu T. (2022). The OsWRKY63-OsWRKY76-OsDREB1B module regulates chilling tolerance in rice. Plant J..

[21] Mao D., Chen C. (2012). Colinearity and similar expression pattern of rice *DREB1s* reveal their functional conservation in the cold-responsive pathway. PLoS ONE.

[22] Zhao C., Lang Z., Zhu J.K. (2015). Cold responsive gene transcription becomes more complex. Trends Plant Sci..

[23] Zhang J., Luo W., Zhao Y., Xu Y., Song S., Chong K. (2016). Comparative metabolomic analysis reveals a reactive oxygen species-dominated dynamic model underlying chilling environment adaptation and tolerance in rice. New Phytol..

[24] Baxter A., Mittler R., Suzuki N. (2014). ROS as key players in plant stress signalling. J. Exp. Bot..

[25] Mittler R., Zandalinas S.I., Fichman Y., Van Breusegem F. (2022). Reactive oxygen species signalling in plant stress responses. Nat. Rev. Mol. Cell Biol..

[26] Li R., Song Y., Wang X., Zheng C., Liu B., Zhang H., Ke J., Wu X., Wu L., Yang R. (2024). OsNAC5 orchestrates OsABI5 to fine-tune cold tolerance in rice. J. Integr. Plant Biol..

[27] Sun Y., Xie Z., Jin L., Qin T., Zhan C., Huang J. (2024). Histone deacetylase OsHDA716 represses rice chilling tolerance by deacetylating OsbZIP46 to reduce its transactivation function and protein stability. Plant Cell.

[28] Liu D., Luo S., Li Z., Liang G., Guo Y., Xu Y., Chong K. (2024). COG3 confers the chilling tolerance to mediate OsFtsH2-D1 module in rice. New Phytol..

[29] Ji L., Zhang Z., Liu S., Zhao L., Li Q., Xiao B., Suzuki N., Burks D.J., Azad R.K., Xie G. (2024). The OsTIL1 lipocalin protects cell membranes from reactive oxygen species damage and maintains the 18:3-containing glycerolipid biosynthesis under cold stress in rice. Plant J..

[30] Liu J., Liu J., He M., Zhang C., Liu Y., Li X., Wang Z., Jin X., Sui J., Zhou W. (2024). OsMAPK6 positively regulates rice cold tolerance at seedling stage via phosphorylating and stabilizing OsICE1 and OsIPA1. Theor. Appl. Genet..

[31] Xia C., Liang G., Chong K., Xu Y. (2023). The COG1-OsSERL2 complex senses cold to trigger signaling network for chilling tolerance in *japonica* rice. Nat. Commun..

[32] Gu S., Zhang Z., Li J., Sun J., Cui Z., Li F., Zhuang J., Chen W., Su C., Wu L. (2023). Natural variation in OsSEC13 HOMOLOG 1 modulates redox homeostasis to confer cold tolerance in rice. Plant Physiol..

[33] Benkova E., Michniewicz M., Sauer M., Teichmann T., Seifertova D., Jurgens G., Friml J. (2003). Local, efflux-dependent auxin gradients as a common module for plant organ formation. Cell.

[34] Zhao Y. (2010). Auxin biosynthesis and its role in plant development. Annu. Rev. Plant Biol..

[35] Lavy M., Estelle M. (2016). Mechanisms of auxin signaling. Development.

[36] Mroue S., Simeunovic A., Robert H.S. (2018). Auxin production as an integrator of environmental cues for developmental growth regulation. J. Exp. Bot..

[37] Waadt R., Seller C.A., Hsu P.K., Takahashi Y., Munemasa S., Schroeder J.I. (2022). Plant hormone regulation of abiotic stress responses. Nat. Rev. Mol. Cell Biol..

[38] Shi Y., Ding Y., Yang S. (2015). Cold signal transduction and its interplay with phytohormones during cold acclimation. Plant Cell Physiol..

[39] Gaveliene V., Novickiene L., Pakalniskyte L. (2013). Effect of auxin physiological analogues on rapeseed (*Brassica napus*) cold hardening, seed yield and quality. J. Plant Res..

[40] Du H., Liu H., Xiong L. (2013). Endogenous auxin and jasmonic acid levels are differentially modulated by abiotic stresses in rice. Front. Plant Sci..

[41] Korasick D.A., Enders T.A., Strader L.C. (2013). Auxin biosynthesis and storage forms. J. Exp. Bot..

[42] Zhang J., Peer W.A. (2017). Auxin homeostasis: The DAO of catabolism. J. Exp. Bot..

[43] Kramer E.M., Ackelsberg E.M. (2015). Auxin metabolism rates and implications for plant development. Front. Plant Sci..

[44] Shibasaki K., Uemura M., Tsurumi S., Rahman A. (2009). Auxin response in *Arabidopsis* under cold stress: Underlying molecular mechanisms. Plant Cell.

[45] Swarup R., Peret B. (2012). AUX/LAX family of auxin influx carriers-an overview. Front. Plant Sci..

[46] Konstantinova N., Korbei B., Luschnig C. (2021). Auxin and root gravitropism: Addressing basic cellular processes by exploiting a defined growth response. Int. J. Mol. Sci..

[47] Petrasek J., Mravec J., Bouchard R., Blakeslee J.J., Abas M., Seifertova D., Wisniewska J., Tadele Z., Kubes M., Covanova M. (2006). PIN proteins perform a rate-limiting function in cellular auxin efflux. Science.

[48] Morris D.A. (1979). The effect of temperature on the velocity of exogenous auxin transport in intact chilling-sensitive and chilling-resistant plants. Planta.

[49] Nadella V., Shipp M.J., Muday G.K., Wyatt S.E. (2006). Evidence for altered polar and lateral auxin transport in the *gravity persistent signal* (*gps*) mutants of *Arabidopsis*. Plant Cell Environ..

[50] Steinmann T., Geldner N., Grebe M., Mangold S., Jackson C.L., Paris S., Galweiler L., Palme K., Jurgens G. (1999). Coordinated polar localization of auxin efflux carrier PIN1 by GNOM ARF GEF. Science.

[51] Geldner N., Anders N., Wolters H., Keicher J., Kornberger W., Muller P., Delbarre A., Ueda T., Nakano A., Jurgens G. (2003). The Arabidopsis GNOM ARF-GEF mediates endosomal recycling, auxin transport, and auxin-dependent plant growth. Cell.

[52] Liu S., Wang J., Wang L., Wang X., Xue Y., Wu P., Shou H. (2009). Adventitious root formation in rice requires OsGNOM1 and is mediated by the OsPINs family. Cell Res..

[53] Ashraf M.A., Rahman A. (2019). Cold stress response in *Arabidopsis thaliana* is mediated by GNOM ARF-GEF. Plant J..

[54] Xu H., Yang X., Zhang Y., Wang H., Wu S., Zhang Z., Ahammed G.J., Zhao C., Liu H. (2022). CRISPR/Cas9-mediated mutation in auxin efflux carrier *OsPIN9* confers chilling tolerance by modulating reactive oxygen species homeostasis in rice. Front. Plant Sci..

[55] Ouyang Q., Zhang Y., Yang X., Yang C., Hou D., Liu H., Xu H. (2023). Overexpression of *OsPIN9* impairs chilling tolerance via disturbing ROS homeostasis in rice. Plants.

[56] Yang C., Wang H., Ouyang Q., Chen G., Fu X., Hou D., Xu H. (2023). Deficiency of auxin efflux carrier *OsPIN1b* impairs chilling and drought tolerance in rice. Plants.

[57] Xu H., Zhang Y., Yang X., Wang H., Hou D. (2022). Tissue specificity and responses to abiotic stresses and hormones of *PIN* genes in rice. Biologia.

[58] Wang J.R., Hu H., Wang G.H., Li J., Chen J.Y., Wu P. (2009). Expression of *PIN* genes in rice (*Oryza sativa* L.): Tissue specificity and regulation by hormones. Mol. Plant.

[59] Miyashita Y., Takasugi T., Ito Y. (2010). Identification and expression analysis of *PIN* genes in rice. Plant Sci..

[60] Lu G., Coneva V., Casaretto J.A., Ying S., Mahmood K., Liu F., Nambara E., Bi Y.M., Rothstein S.J. (2015). *OsPIN5b* modulates rice (*Oryza sativa*) plant architecture and yield by changing auxin homeostasis, transport and distribution. Plant J..

[61] Liu Q., Chen T.T., Xiao D.W., Zhao S.M., Lin J.S., Wang T., Li Y.J., Hou B.K. (2019). *OsIAGT1* is a glucosyltransferase gene involved in the glucose conjugation of auxins in rice. Rice.

[62] Sang D., Chen D., Liu G., Liang Y., Huang L., Meng X., Chu J., Sun X., Dong G., Yuan Y. (2014). Strigolactones regulate rice tiller angle by attenuating shoot gravitropism through inhibiting auxin biosynthesis. Proc. Natl. Acad. Sci. USA.

[63] Zhang N., Yu H., Yu H., Cai Y., Huang L., Xu C., Xiong G., Meng X., Wang J., Chen H. (2018). A core regulatory pathway controlling rice tiller angle mediated by the *LAZY1*-dependent asymmetric distribution of auxin. Plant Cell.

[64] Li Z., Liang Y., Yuan Y.D., Wang L., Meng X.B., Xiong G.S., Zhou J., Cai Y.Y., Han N.P., Hua L.K. (2019). OsBRXL4 regulates shoot gravitropism and rice tiller angle through affecting LAZY1 nuclear localization. Mol. Plant.

[65] Staswick P.E., Serban B., Rowe M., Tiryaki I., Maldonado M.T., Maldonado M.C., Suza W. (2005). Characterization of an Arabidopsis enzyme family that conjugates amino acids to indole-3-acetic acid. Plant Cell.

[66] Fu J., Yu H., Li X., Xiao J., Wang S. (2011). Rice GH3 gene family: Regulators of growth and development. Plant Signal Behav..

[67] Du H., Wu N., Fu J., Wang S., Li X., Xiao J., Xiong L. (2012). A GH3 family member, OsGH3-2, modulates auxin and abscisic acid levels and differentially affects drought and cold tolerance in rice. J. Exp. Bot..

[68] Abraham E., Rigo G., Szekely G., Nagy R., Koncz C., Szabados L. (2003). Light-dependent induction of proline biosynthesis by abscisic acid and salt stress is inhibited by brassinosteroid in *Arabidopsis*. Plant Mol. Biol..

[69] Sun S.J., Guo S.Q., Yang X., Bao Y.M., Tang H.J., Sun H., Huang J., Zhang H.S. (2010). Functional analysis of a novel Cys2/His2-type zinc finger protein involved in salt tolerance in rice. J. Exp. Bot..

[70] Wang Y., Zhang M., Wu C., Chen C., Meng L., Zhang G., Zhuang K., Shi Q. (2024). SlWRKY51 regulates proline content to enhance chilling tolerance in tomato. Plant Cell Environ..

[71] Hu C.H., Wang P.Q., Zhang P.P., Nie X.M., Li B.B., Tai L., Liu W.T., Li W.Q., Chen K.M. (2020). NADPH oxidases: The vital performers and center hubs during plant growth and signaling. Cells.

[72] Marino D., Dunand C., Puppo A., Pauly N. (2012). A burst of plant NADPH oxidases. Trends Plant Sci..

[73] Zhao X.Y., Wang H.Q., Shi W., Zhang W.W., Zhao F.J. (2025). The respiratory burst oxidase homologue OsRBOHE is crucial for root hair formation, drought resistance and tillering in rice. Plant Cell Environ..

[74] Wong H.L., Pinontoan R., Hayashi K., Tabata R., Yaeno T., Hasegawa K., Kojima C., Yoshioka H., Iba K., Kawasaki T. (2007). Regulation of rice NADPH oxidase by binding of Rac GTPase to its N-terminal extension. Plant Cell.

[75] Keller T., Damude H.G., Werner D., Doerner P., Dixon R.A., Lamb C. (1998). A plant homolog of the neutrophil NADPH oxidase gp91*^phox^* subunit gene encodes a plasma membrane protein with Ca^2+^ binding motifs. Plant Cell.

[76] Yamauchi T., Yoshioka M., Fukazawa A., Mori H., Nishizawa N.K., Tsutsumi N., Yoshioka H., Nakazono M. (2017). An NADPH oxidase RBOH functions in rice roots during lysigenous aerenchyma formation under oxygen-deficient conditions. Plant Cell.

[77] Ding Y., Shi Y., Yang S. (2024). Regulatory networks underlying plant responses and adaptation to cold stress. Annu. Rev. Genet..

[78] Kidokoro S., Shinozaki K., Yamaguchi-Shinozaki K. (2022). Transcriptional regulatory network of plant cold-stress responses. Trends Plant Sci..

[79] Aslam M., Fakher B., Ashraf M.A., Cheng Y., Wang B.R., Qin Y. (2022). Plant low-temperature stress: Signaling and response. Agronomy.

[80] Xu M., Zhu L., Shou H., Wu P. (2005). A *PIN1* family gene, *OsPIN1*, involved in auxin-dependent adventitious root emergence and tillering in rice. Plant Cell Physiol..

[81] Li Y., Zhu J., Wu L., Shao Y., Wu Y., Mao C. (2019). Functional divergence of *PIN1* paralogous genes in rice. Plant Cell Physiol..

[82] Wang L., Guo M., Li Y., Ruan W., Mo X., Wu Z., Sturrock C.J., Yu H., Lu C., Peng J. (2018). *LARGE ROOT ANGLE1*, encoding OsPIN2, is involved in root system architecture in rice. J. Exp. Bot..

[83] Inahashi H., Shelley I.J., Yamauchi T., Nishiuchi S., Takahashi-Nosaka M., Matsunami M., Ogawa A., Noda Y., Inukai Y. (2018). *OsPIN2*, which encodes a member of the auxin efflux carrier proteins, is involved in root elongation growth and lateral root formation patterns via the regulation of auxin distribution in rice. Physiol. Plant.

[84] Li W.Q., Zhang M.J., Qiao L., Chen Y.B., Zhang D.P., Jing X.Q., Gan P.F., Huang Y.B., Gao J.R., Liu W.T. (2022). Characterization of *wavy root 1*, an agravitropism allele, reveals the functions of *OsPIN2* in fine regulation of auxin transport and distribution and in ABA biosynthesis and response in rice (*Oryza sativa* L.). Crop J..

[85] Hao B., Zhang R., Zhang C., Wen N., Xia Y., Zhao Y., Li Q., Qiao L., Li W. (2024). Characterization of *OsPIN2* mutants reveal novel roles for reactive oxygen species in modulating not only root gravitropism but also hypoxia tolerance in rice seedlings. Plants.

[86] Zeng Y., Wen J., Zhao W., Wang Q., Huang W. (2019). Rational Improvement of Rice Yield and Cold Tolerance by Editing the Three Genes OsPIN5b, GS3, and OsMYB30 with the CRISPR-Cas9 System. Front. Plant Sci..

[87] Han X.L., Zhao F.Y. (2022). OsYUCCA2 deficiency in rice growth and development. Cienc. Rural..

[88] Xu X.Y., Zhiguo E., Zhang D.P., Yun Q.B., Zhou Y., Niu B.X., Chen C. (2021). *OsYUC11*-mediated auxin biosynthesis is essential for endosperm development of rice. Plant Physiol..

[89] Zhao Z., Zhang Y., Liu X., Zhang X., Liu S., Yu X., Ren Y., Zheng X., Zhou K., Jiang L. (2013). A role for a dioxygenase in auxin metabolism and reproductive development in rice. Dev. Cell.

[90] Hou M., Luo F., Wu D., Zhang X., Lou M., Shen D., Yan M., Mao C., Fan X., Xu G. (2021). OsPIN9, an auxin efflux carrier, is required for the regulation of rice tiller bud outgrowth by ammonium. New Phytol..

[91] Suzuki N., Koussevitzky S., Mittler R., Miller G. (2012). ROS and redox signalling in the response of plants to abiotic stress. Plant Cell Environ..

[92] Liu H., Song S., Zhang H., Li Y., Niu L., Zhang J., Wang W. (2022). Signaling transduction of ABA, ROS, and Ca^2+^ in plant stomatal closure in response to drought. Int. J. Mol. Sci..

[93] Huang H., Ullah F., Zhou D.X., Yi M., Zhao Y. (2019). Mechanisms of ROS regulation of plant development and stress responses. Front. Plant Sci..

[94] Sun M., Shen Y., Chen Y., Wang Y., Cai X., Yang J., Jia B., Dong W., Chen X., Sun X. (2022). *Osa-miR1320* targets the ERF transcription factor OsERF096 to regulate cold tolerance via JA-mediated signaling. Plant Physiol..

[95] Ben Rejeb K., Abdelly C., Savouré A. (2014). How reactive oxygen species and proline face stress together. Plant Physiol. Biochem..

[96] Zhao Z.F., Sun A.Q., Shan W.F., Zheng X.H., Wang Y., Bai L., Xu Y.C., An Z., Wang X.Y., Wang Y.M. (2024). OsRbohI is the indispensable NADPH oxidase for molecular-patterns-induced reactive oxygen species production in rice. Plant Commun..

[97] Xu H.W., Ji X.M., He Z.H., Shi W.P., Zhu G.H., Niu J.K., Li B.S., Peng X.X. (2006). Oxalate accumulation and regulation is independent of glycolate oxidase in rice leaves. J. Exp. Bot..

[98] Hiei Y., Ohta S., Komari T., Kumashiro T. (1994). Efficient transformation of rice (*Oryza sativa* L.) mediated by *Agrobacterium* and sequence analysis of the boundaries of the T-DNA. Plant J..

[99] Manna M., Rengasamy B., Sinha A.K. (2024). A rapid and robust colorimetric method for measuring relative abundance of auxins in plant tissues. Phytochem. Anal..

[100] Choi H.W., Kim Y.J., Lee S.C., Hong J.K., Hwang B.K. (2007). Hydrogen peroxide generation by the pepper extracellular peroxidase CaPO2 activates local and systemic cell death and defense response to bacterial pathogens. Plant Physiol..

[101] Hu Z., Huang X., Amombo E., Liu A., Fan J., Bi A., Ji K., Xin H., Chen L., Fu J. (2020). The ethylene responsive factor CdERF1 from bermudagrass (*Cynodon dactylon*) positively regulates cold tolerance. Plant Sci..

[102] Morris D.L. (1948). Quantitative determination of carbohydrates with dreywood’s anthrone reagent. Science.

[103] Troll W., Lindsley J. (1955). A photometric method for the determination of proline. J. Biol. Chem..

[104] Wu F.B., Zhang G.P., Dominy P. (2003). Four barley genotypes respond differently to cadmium: Lipid peroxidation and activities of antioxidant capacity. Environ. Exp. Bot..

[105] Bradford M.M. (1976). A rapid and sensitive method for the quantitation of microgram quantities of protein utilizing the principle of protein-dye binding. Anal. Biochem..

