# Supplementary material for: Overexpression of OsPIN5b Alters Plant Architecture and Impairs Cold Tolerance in Rice (Oryza sativa L.)
Source: Plants (Basel). 2025 Mar 25;14(7):1026. doi: 10.3390/plants14071026 (PMC11990878; doi:10.3390/plants14071026)

Figure S1. Phenotypes of wild-type (WT) and OsPIN5b-overexpressing lines (OE) at 7 days (A) and 14 days (B) post-germination. Bar = 5 cm. Values are means ± standard deviation (SD; n = 30). Data were analyzed by ANOVA and Tukey's tests at $p < 0.05$ significant level. *: $p < 0.05$; **: $p < 0.01$; ***: $p < 0.001$.

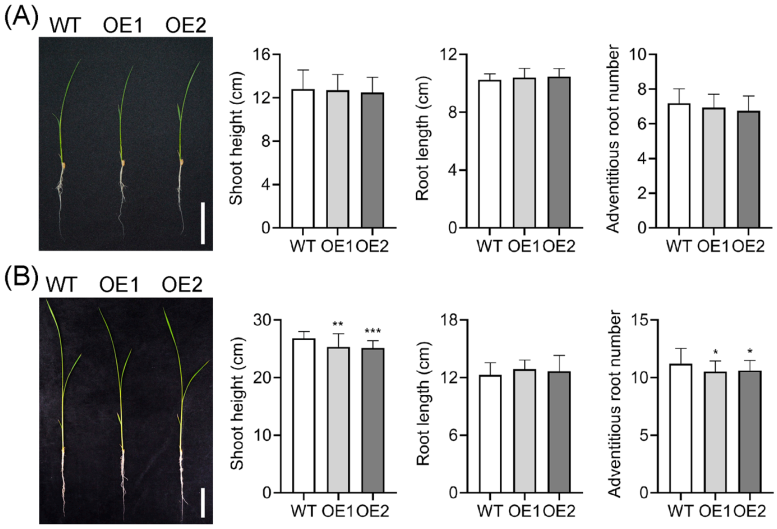

Supplement: Supplementary file 1 [file plants-14-01026-s001.zip › Supplementary files-Figure S1.pdf]
