# Supplementary material for: Overexpression of OsPIN5b Alters Plant Architecture and Impairs Cold Tolerance in Rice (Oryza sativa L.)
Source: Plants (Basel). 2025 Mar 25;14(7):1026. doi: 10.3390/plants14071026 (PMC11990878; doi:10.3390/plants14071026)

Figure S4. Expression analysis of *GH3* genes in WT and OE leaves (A) and roots (B). Values are means ± standard deviation (SD; n = 3). Data were analyzed by ANOVA and Tukey's tests at $p < 0.05$ significant level. *: $p < 0.05$; ***: $p < 0.001$.

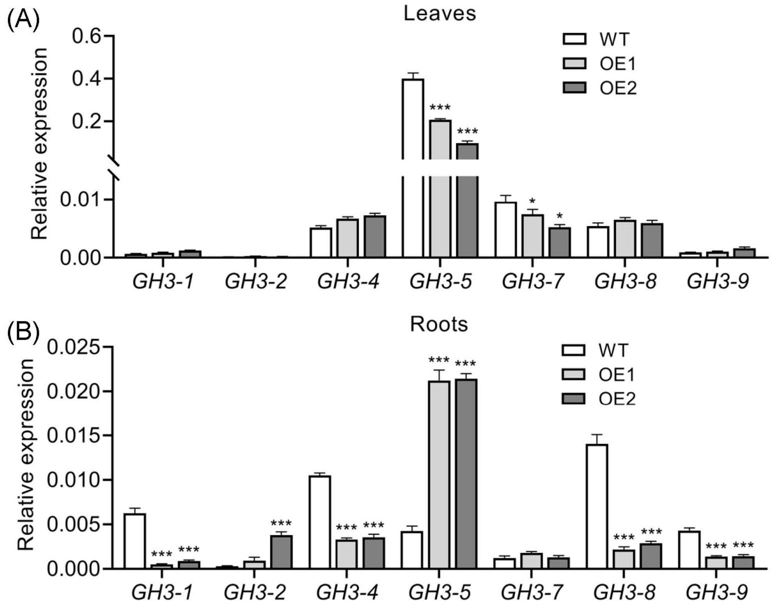

Supplement: Supplementary file 1 [file plants-14-01026-s001.zip › Supplementary files-Figure S4.pdf]
