# Supplementary material for: Overexpression of OsPIN5b Alters Plant Architecture and Impairs Cold Tolerance in Rice (Oryza sativa L.)
Source: Plants (Basel). 2025 Mar 25;14(7):1026. doi: 10.3390/plants14071026 (PMC11990878; doi:10.3390/plants14071026)
Supplement: Supplementary file 1 [file plants-14-01026-s001.zip › Supplementary files-Table S1.pdf]

Table S1. Primers used in this study.

**Primers for vector construction**	Primer sequences (5′-3′)
OsPIN5b-F	tgtttggtgttacttctgcagATGATAGGATGGGGAGATGTGTACAAGGT
OsPIN5b-R	gcccttgctcaccatggatccGACAAAGCCCAGAACCGCGTAATATG
**Primers for screening of the transgenic plants**	
HPT-F	CTGAACTCACCGCGACGTCTGTC
HPF-R	TAGCGCGTCTGCTGCTCCATACA
**Primers for qRT-PCR**	
OsPIN1a-qF	CCTGAAATCCATCTCCATCCTC
OsPIN1a-qR	AACGTCGCCACCTTGTT
OsPIN1b-qF	GTCTTCGCCAAGGAGTACAG
OsPIN1b-qR	TGTAGTAGACGAGGGTGATAGG
OsPIN1c-qF	GAATCGTGCCCTTTGTGTTTG
OsPIN1c-qR	GAGCAATCAGCATCCCGAATA
OsPIN2-qF	CGTCTCCTTCAGGTGGAATATC
OsPIN2-qR	AGAGCCATGAACAAGCCTAAG
OsPIN5a-qF	CCCTACCTCAATCCATCACATC
OsPIN5a-qR	GTAGGGAGACAAGCATTCCAA
OsPIN5b-qF	GCAAAGGAGTATGGGCTTCA
OsPIN5b-qR	GCAATCAGAATCGGCAGAGA
OsPIN5c-qF	GACACAAGTCCTCACGATGAA
OsPIN5c-qR	TAACCGCTGTGCTGAGTATTT
OsPIN9-qF	GAGGACTCTCTGTTCACCATTC
OsPIN9-qR	GAGAACGACGCTATCTTGTATCC
OsYUC1-qF	AGGTGTTGGTCGTGGGATGCG
OsYUC1-qR	GCGATGCCGAACGTGGATAGA
OsYUC3-qF	GGAAGCGTGTTCTCGTTGTTG
OsYUC3-qR	ACATTGACAGCCCAAAGGTGG
OsYUC4-qF	CCTCGACCTCTGCAACCACAATG
OsYUC4-qR	CGACAACAGGAGTACCAGCCAATC
OsYUC6-qF	GGATACCAAAGCAACGTCCCC
OsYUC6-qR	TGAAGCCAACAGAGTAGAGCCCTG
OsYUC7-qF	ACCGGCTACCGCAGCAATGTG
OsYUC7-qR	CGTACAGCCCCGACTCACCCT
OsYUC8-qF	GAGATGTGCCTGGACCTCTGC
OsYUC8-qR	GTGTCTCCCAGCACCATCCTT
OsIAA20-qF	TGGCGGATATGTGAAGGTGAA
OsIAA20-qR	TATGAGCCGAGGATGGACAAG
GH3.1-qF	ATCGCCGACGAGATGAACAG

GH3.1-qR	GGCTCCGGTAGTAGCTTGTG
GH3.2-qF	GGGGAGAGGAAGCTAATGCC
GH3.2-qR	CACGTTGTAGGGGTCGAAGG
GH3.4-qF	CCACCTACTTCAGCCCCAAG
GH3.4-qR	GCCAAGCTATCACAGGTCGT
GH3.5-qF	GGACTGATGCCGAGAGCTAC
GH3.5-qR	CTTGGGCTTTCCGTGTGTTG
GH3.7-qF	GATAAGTGCACAAGCCCCCT
GH3.7-qR	CGCAGAGGAGTATGCGTGAT
GH3.8-qF	GTATGTGCCTGGGCTTGACA
GH3.8-qR	TGTAGTAGCTCGTCAGCACG
GH3.9-qF	ACGAGCGTCTACAAAAGCGA
GH3.9-qR	CCCAGTTGAGCTGGAGGAAG
OsRbohA-qF	AGAACTGTTTTCTCTGAGGC
OsRbohA-qR	AAGTTTTGGGAATCTTGCTT
OsRbohB-qF	GAAATGCACAACGAAGTTCGAA
OsRbohB-qR	TAACTATATGCTTGCTTCCTCA
OsRbohC-qF	TACACTCACGAAACAACTCAAGG
OsRbohC-qR	TATTTGCTATCCCAATGCTATCGT
OsRbohD-qF	GCAGCAAGATCCGGACACAT
OsRbohD-qR	GAACCGAGTCGTTGTCGTCT
OsRbohE-qF	CAGATGCACGTCAAAATTTGAG
OsRbohE-qR	CTGAGTATCTCAAGCAAGAAGT
OsRbohF-qF	AAAGCCCACACTAGCCAAAGAAT
OsRbohF-qR	AGAGGGTCAGTTTGCTTCTATGC
OsRbohG-qF	AACAGTTGGAGTTTTCTACTGC
OsRbohG-qR	TGGAGATCAGGAGAAGCTATATC
OsRbohH-qF	AGCTCGCTCAAGATTTCTCGAG
OsRbohH-qR	TTGATCTAATCCCTCTACATTTCCA
OsRbohI-qF	TCATCGGATTCATCGTCGTC
OsRbohI-qR	CGAACAACCAATCACTCACT
OsACTIN1-qF	CTTCATAGGAATGGAAGCTGCG
OsACTIN1-qR	CACCTTGATCTTCATGCTGCTA
