# Supplementary material for: Overexpression of OsPIN5b Alters Plant Architecture and Impairs Cold Tolerance in Rice (Oryza sativa L.)
Source: Plants (Basel). 2025 Mar 25;14(7):1026. doi: 10.3390/plants14071026 (PMC11990878; doi:10.3390/plants14071026)
Supplement: Supplementary file 1 [file plants-14-01026-s001.zip › Supplementary files-Table S2.pdf]

Table S2. Gene name and ID numbers used for qRT-PCR in this study.

Gene name	Gene ID
OsPIN1a	Os06g0232300
OsPIN1b	Os02g0743400
OsPIN1c	Os11g0137000
OsPIN2	Os06g0660200
OsPIN5a	Os01g0919800
OsPIN5b	Os08g0529000
OsPIN5c	Os09g0505400
OsPIN9	Os01g0802700
OsYUC1	Os01g0645400
OsYUC3	Os01g0732700
OsYUC4	Os01g0224700
OsYUC6	Os07g0437000
OsYUC7	Os04g0128900
OsYUC8	Os03g0162000
OsIAA20	Os06g0166500
GH3-1	Os01g0785400
GH3-2	Os01g0764800
GH3-4	Os05g0500900
GH3-5	Os05g0586200
GH3-7	Os06g0499500
GH3-8	Os07g0592600
GH3-9	Os07g0576500
OsRbohA	Os01g0734200
OsRbohB	Os01g0360200
OsRbohC	Os05g0528000
OsRbohD	Os05g0465800
OsRbohE	Os01g0835500
OsRbohF	Os08g0453700
OsRbohG	Os09g0438000
OsRbohH	Os12g0541300
OsRbohI	Os11g0537400
OsACTIN1	Os03g0718100
